# A multi-modal multi-branch framework for retinal vessel segmentation using ultra-widefield fundus photographs

**DOI:** 10.3389/fcell.2024.1532228

**Published:** 2025-01-08

**Authors:** Qihang Xie, Xuefei Li, Yuanyuan Li, Jiayi Lu, Shaodong Ma, Yitian Zhao, Jiong Zhang

**Affiliations:** ^1^ Cixi Biomedical Research Institute, Wenzhou Medical University, Ningbo, China; ^2^ Laboratory of Advanced Theranostic Materials and Technology, Ningbo Institute of Materials Technology and Engineering, Chinese Academy of Sciences, Ningbo, China

**Keywords:** ultra-widefield, fundus fluorescence angiography, retinal vessel segmentation, multimodal framework, selective fusion

## Abstract

**Background:**

Vessel segmentation in fundus photography has become a cornerstone technique for disease analysis. Within this field, Ultra-WideField (UWF) fundus images offer distinct advantages, including an expansive imaging range, detailed lesion data, and minimal adverse effects. However, the high resolution and low contrast inherent to UWF fundus images present significant challenges for accurate segmentation using deep learning methods, thereby complicating disease analysis in this context.

**Methods:**

To address these issues, this study introduces M3B-Net, a novel multi-modal, multi-branch framework that leverages fundus fluorescence angiography (FFA) images to improve retinal vessel segmentation in UWF fundus images. Specifically, M3B-Net tackles the low segmentation accuracy caused by the inherently low contrast of UWF fundus images. Additionally, we propose an enhanced UWF-based segmentation network in M3B-Net, specifically designed to improve the segmentation of fine retinal vessels. The segmentation network includes the Selective Fusion Module (SFM), which enhances feature extraction within the segmentation network by integrating features generated during the FFA imaging process. To further address the challenges of high-resolution UWF fundus images, we introduce a Local Perception Fusion Module (LPFM) to mitigate context loss during the segmentation cut-patch process. Complementing this, the Attention-Guided Upsampling Module (AUM) enhances segmentation performance through convolution operations guided by attention mechanisms.

**Results:**

Extensive experimental evaluations demonstrate that our approach significantly outperforms existing state-of-the-art methods for UWF fundus image segmentation.

## 1 Introduction

Clinical studies have demonstrated that alterations in the morphology of fundus blood vessels are closely associated with the progression of ocular diseases. By analyzing these vascular changes, physicians can diagnose and evaluate the severity and nature of various eye conditions. Ultra-WideField (UWF) imaging, an advanced form of fundus imaging, has emerged as a critical tool for disease analysis. With its expansive 200-degree field of view, UWF imaging provides a significantly broader depiction of vascular and disease-related features compared to conventional color fundus imaging, which is typically limited to a 45-degree range. Fundus fluorescein angiography (FFA) images are regarded as the gold standard for clinical vascular detection due to their superior contrast and precise diagnostic information. However, FFA imaging is invasive and carries potential side effects, limiting its broader applicability. In contrast, UWF imaging is a non-invasive modality that offers high-resolution visualization of fundus structures across a panoramic field of view exceeding 200°. This technology enables detailed detection and analysis of peripheral retinal vessels and focal areas of pathology, such as those seen in diabetic retinopathy and venous obstruction. UWF fundus imaging excels in capturing intricate views of peripheral retinal vessels and lesions, providing rich pathological information that significantly enhances clinical analysis (Nguyen et al., 2024; Liu et al., 2023; Chen et al., 2023; Zhang et al., 2021). In computational fundus image analysis, vascular segmentation stands out as a foundational and extensively studied task. Accurate delineation of fundus vascular structures is essential, serving as a prerequisite for effective disease diagnosis and assessment (Yang et al., 2023).

Over the past decade, significant progress has been made in fundus vessel segmentation, driven by advancements in filtering (Zhang et al., 2016; Soares et al., 2006; Memari et al., 2017), morphological (Fraz et al., 2013; Imani et al., 2015; Kumar and Ravichandran, 2017), statistical (Orlando et al., 2016; Yin et al., 2012; Oliveira et al., 2018), and deep learning algorithms (Wu et al., 2018; Yan et al., 2018; Ryu et al., 2023). Among these, deep learning techniques have demonstrated particularly remarkable potential in medical image processing. The Key contributions include the window-based and sliding window approaches for neural cell membrane segmentation in microscopy images proposed by Ciresan et al. (2012), and the integration of multi-scale 3D Convolutional Neural Networks (CNNs) with Conditional Random Fields (CRFs) for brain lesion segmentation presented by Kamnitsas et al. (2017). The introduction of end-to-end Fully Convolutional Networks (FCNs) (Long et al., 2015) marked a transformative milestone in biomedical image segmentation. Among these, U-Net, an iconic encoder-decoder architecture introduced by Ronneberger et al. (2015), has gained widespread recognition for its ability to effectively integrate multi-resolution features. U-Net’s superior performance in medical image segmentation has spurred the development of numerous refined iterations and enhanced versions (Fu et al., 2016; Gibson et al., 2018). To further improve segmentation performance, several multi-stage models have been proposed. For instance (Yan et al., 2018), developed a three-stage deep learning model for fundus blood vessel segmentation. Additionally, weakly supervised and semi-supervised approaches have been explored to address the challenge of sparsely labeled medical image data (Perone and Cohen-Adad, 2018). extended the Mean Teacher method for MRI segmentation, while (Seeböck et al., 2019) proposed a Bayesian U-Net for abnormality detection in OCT image segmentation. Despite these advancements, challenges such as segmentation inaccuracies and limited adaptability persist. Moreover, relatively few studies have focused on Ultra-WideField imaging modalities, underscoring the need for further exploration in this domain.

Compared to color fundus images, limited research has focused on the extraction of integrated vascular structures in Ultra-WideField (UWF) fundus images. There has been research focused on the analysis and diagnosis of ophthalmic diseases based on UWF images (Tang et al., 2024; Deng et al., 2024; Wan et al., 2024) proposed a new segmentation algorithm for peripapillary atrophy and optic disk from UWF Photographs (Ding et al., 2020). introduced a deep-learning framework for efficient vascular detection in UWF fundus photography (Ong et al., 2023). employed U-Net (Ronneberger et al., 2015) to segment UWF fundus images for recognizing lesions associated with retinal vein occlusion. Recently (Wang et al., 2024), developed a pioneering framework utilizing a patch-based active domain adaptation approach to enhance vessel segmentation in UWF-SLO images by selectively identifying valuable image patches. Additionally, they constructed and annotated the first multi-center UWF-SLO vessel segmentation dataset, incorporating data from multiple institutions to advance research in this area. Similarly (Wu et al., 2024), released the first publicly available UWF retinal hemorrhage segmentation dataset and proposed a subtraction network specifically for UWF retinal hemorrhage segmentation (Ju et al., 2021). explored a modified Cycle Generative Adversarial Network (CycleGAN) (Zhu et al., 2017) to bridge the domain gap between standard and UWF fundus images, facilitating the training of UWF fundus diagnosis models. Despite these advancements, feature extraction for UWF fundus images remains underdeveloped, and the accuracy of blood vessel segmentation in UWF fundus images requires further improvement.

The study of blood vessel segmentation in UWF fundus images presents several complex challenges. Firstly, one of the primary challenges is the complex backgrounds and uneven illumination characteristic of UWF fundus images, which result in low contrast between blood vessels and surrounding tissue, making feature extraction more difficult. In contrast, Fundus Fluorescence Angiography (FFA) images offer substantially higher contrast for blood vessels, enabling more precise extraction of vascular features. This distinction highlights the potential of using FFA images as high-quality references to improve the accuracy of vascular feature extraction in UWF fundus images. In this context, style transfer—an unsupervised image generation technique—has emerged as a powerful tool for image style conversion and enhancement. Owing to its flexibility and effectiveness, style transfer algorithms are increasingly employed across a range of multimodal medical imaging tasks.

For example (Tavakkoli et al., 2020), introduced a deep learning conditional GAN capable of generating FFA images from fundus photographs. Their proposed GAN produced anatomically accurate angiograms with fidelity comparable to FFA images, significantly outperforming two state-of-the-art generative algorithms. Similarly (Ju et al., 2021), employed an enhanced CycleGAN (Zhu et al., 2017) to facilitate modal shifts between standard fundus images and UWF fundus images, effectively generating additional UWF fundus images for training purposes. This approach addressed challenges related to data scarcity and annotation. Inspired by these advancements in leveraging style transfer for downstream tasks, our research explores cross-modality-assisted feature extraction in UWF fundus images using style transfer models. Specifically, this thesis proposes a style transfer model to optimize the extraction of critical blood vessel features during the conversion from UWF to FFA images. This methodology aims to enhance the accuracy of retinal vessel segmentation in UWF fundus images.

Feature selection involves eliminating redundant or irrelevant features from a set of extracted features to improve performance. Recent advancements in image super-resolution have showcased the enhancement of low-resolution (LR) images by aligning LR and reference images in the feature space and fusing them using deep architectures. For instance (Wan et al., 2022), proposed a novel ternary translation network that transforms aged and pristine photos into a shared latent space. Pairwise learning is employed to translate between these latent spaces, generating quality-enhanced photos. Furthermore, several studies have utilized semantic consistency across diverse images as a form of bootstrapping during training, achieved by computing feature similarity (Wu et al., 2023). Inspired by these advancements, we explore search matching within the feature space for feature selection in a multi-stream framework. By identifying key features in UWF fundus images within the segmentation network, our approach achieves more precise segmentation of low-contrast retinal vessels.

Secondly, in addition to the challenge of low contrast, the high-resolution nature of UWF imaging poses a significant limitation to segmentation accuracy. Computational constraints have led many recent studies to adopt a patch-based approach for segmenting images (Wang et al., 2024). However, this method often disregards the interaction between local patches and the global context, resulting in segmentation inaccuracies, particularly in delineating edge details. To address this limitation, recent research has advocated for combining global and local features through multi-stream networks to enhance contextual awareness. These approaches have successfully mitigated the loss of contextual information inherent in patch-based segmentation methods. Building on these advancements, we propose incorporating a local-aware context module into the retinal vessel segmentation network for UWF images. This module aims to overcome the issue of blood vessel fragmentation by effectively integrating global and local information, thereby improving segmentation accuracy for high-resolution UWF fundus images.

Our contributions are summarized as follows:

• We propose a multi-modal, multi-branch framework, named M3B-Net, which is based on a style transfer strategy. This framework leverages the conversion of UWF fundus images to FFA images to enhance segmentation tasks for UWF fundus images. M3B-Net comprises two primary components: a style transfer network and a segmentation network.

• we incorporate a Selective Fusion Module (SFM) into the segmentation network to address the challenges of low contrast in UWF image segmentation. The SFM performs feature space matching to extract key features in the latent space, mitigating issues such as blood vessel fragmentation.

• We introduce a multi-scale local perception fusion module (LPFM) into the segmentation network according to the high-resolution nature of UWF fundus images. This module enhances the network’s ability to discern contextual information effectively.

• We propose a multi-level attention upsampling module (AUM) to improve the segmentation of fine blood vessels. This module aims to mitigate feature loss during the upsampling process, enabling more precise segmentation of delicate vascular sr = tryctures in UWF fundus images.

## 2 Methods

### 2.1 Dataset

In this work, we validate the efficacy of our method using two datasets: a proprietary dataset and the publicly available PRIME-FP20 dataset (Ding et al., 2020).

**FIGURE 1:**
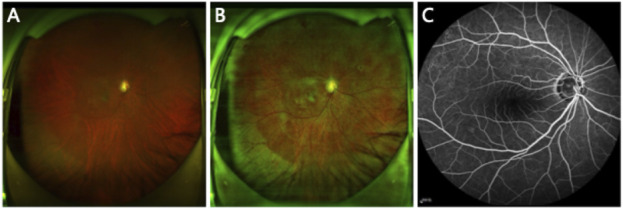
**(A)** Original UWF image. **(B)** Preprocessed UWF image. **(C)** FFA image paired with the UWF image.

• The private dataset used in this study was collected at Ningbo Yinzhou People’s Hospital. The fundus UWF image dataset was captured using an Ultra-WideField Laser Scanning Ophthalmoscope of Optos 200Tx with a resolution of 3900×3072. The paired FFA images were obtained by a Heidelberg Spectralis HRA Instrument of the same unit, with a resolution of 768×868. Based on this data, we constructed a UWF-FFA dataset (UWF-GAN) and a dataset for segmentation (UWF-SEG). The UWF-GAN dataset consists of 120 aligned UWF image patches and FFA image patches, which are intercepted and aligned from the paired whole UWF fundus images and FFA images. The UWF-SEG dataset comprises 65 UWF fundus images, each annotated with pixel-level vessel labels for evaluating segmentation performance. The vessel labels of the UWF fundus images were annotated by an experienced ophthalmologist using the Pair annotation tool. All images were collected with explicit patient consent, and we rigorously adhered to data security regulations by meticulously anonymizing all personal information to ensure patient privacy adequately. Figure 1 illustrates examples of the original UWF fundus images, the preprocessed UWF images, and the corresponding paired FFA image. We utilize histogram equalization to preprocess UWF fundus images, effectively highlighting the vascular structures. Both our method and the comparison methods use the preprocessed images as input.

• The PRIME-FP20 dataset was designed to support the development and evaluation of retinal vessel segmentation algorithms for UWF fundus images. It contains 15 high-resolution UWF fundus images acquired using the Optos 200Tx camera (Optos plc, Dunfermline, United Kingdom). Each image is paired with a labeled binarized vascular map and a binarized mask that delineates the effective data area within the image. Additionally, a corresponding FFA image was simultaneously captured for each UWF fundus image. All images in the dataset feature a resolution of 4000×4000 pixels.

### 2.2 Framework overview

**FIGURE 2:**
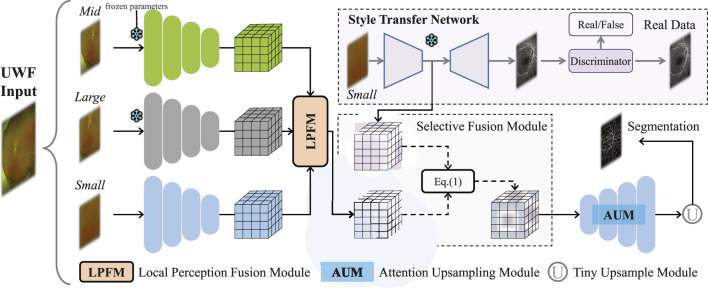
The proposed framework M3B-Net for blood vessel segmentation in UWF fundus images.

The proposed framework is illustrated in Figure 2, comprising two main components: a segmentation network for UWF fundus images and a style transfer network that generates FFA images from UWF fundus images. The segmentation network includes an encoder with a Local Perception Fusion Module (LPFM), a Selective Fusion Module (SFM), a decoder equipped with a Multi-level Attention Upsampling Module (AUM), and an auxiliary tiny encoder-decoder structure. Meanwhile, the style transfer network utilizes a CycleGAN-based architecture (Zhu et al., 2017).

Our method consists of four stages. Stage 1: Use small patches to pre-train a transfer network to obtain FFA features. Stage 2: Use middle patches to pre-train an encoder, aiming to reduce computational overhead during segmentation network training. Stage 3: Similarly, large patches are used to pre-train a large-scale encoder. Stage 4: Freeze the network parameters from the first three stages and begin training the entire segmentation network. In the fourth stage, UWF fundus images X from the dataset are simultaneously fed into both the segmentation network and the style transfer network initially. In the segmentation network, the LPFM processes the UWF images, extracting a feature map $Z_{1}$ . This feature map is passed through the decoder with the multi-level AUM and subsequently processed by a compact U-shaped network to produce the final blood vessel segmentation results. Concurrently, in the CycleGAN-based style transfer network, the UWF images undergo encoding and decoding, resulting in another feature map $Z_{2}$ . During the training phase, $Z_{1}$ and $Z_{2}$ are subjected to a search and matching process via the SFM, which aligns the feature maps from both networks. This alignment establishes a correspondence between the UWF and FFA images. Supervision for both networks is provided through a loss function based on paired UWF and FFA image biplets. The SFM filters the most relevant $Z_{1}$ , which is subsequently fed into the downstream segmentation framework. The following sections provide detailed descriptions of the vessel segmentation network, the Selective Fusion Module, and the Multi-level Attention Upsampling Module.

### 2.3 Vessel segmentation network

The large size of UWF fundus images presents a challenge in balancing segmentation accuracy and computational efficiency for blood vessel segmentation networks. Traditional methods, such as down-sampling, patch cropping, and cascade modeling, struggle to achieve this balance effectively. In this paper, we propose an innovative cropping strategy to address this issue. We utilize an efficient approach by randomly extracting smaller patches from the original UWF image (size 3900×3072 ), resulting in patches of size 256×256 , referred to as ‘small patches’. From these small patches, we generate medium and large patches with dimensions of 512×512 and 768×768 , respectively. Centered on the small patch, we expand it by a factor of two to obtain a middle patch of 512×512 , and similarly, expand it by a factor of three to obtain a large patch of 768×768 . All three patch sizes are then resized to a uniform size of 256×256 and simultaneously input into three ResNet (He et al., 2016) feature extractors. The feature extractors for the medium and large patches are pre-trained, and their parameters are fixed during the segmentation process. This results in three distinct sets of feature maps corresponding to different scales.

**FIGURE 3:**
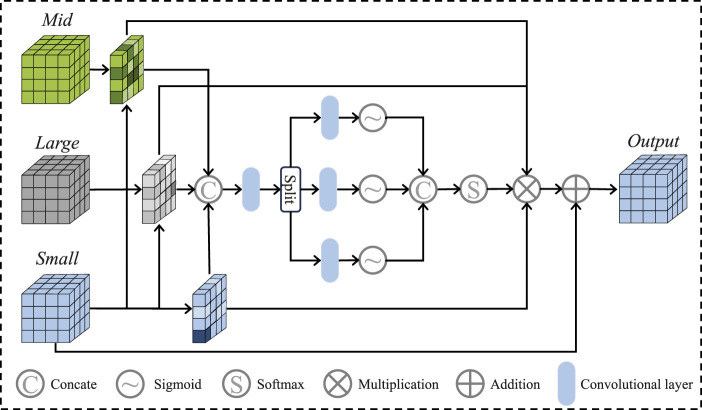
The details of the local perception fusion module framework.

After that, we apply the Local Perception Fusion Module (LPFM) to enhance the contextual positional features of the small patches across different scales. The process of the LPFM is illustrated in Figure 3. First, we compute the inner products between the small patch and both the medium and large patches, obtaining attention maps through softmax activation. These attention maps are then used to generate new feature maps by performing inner product operations with the small patches. The resulting feature maps are regularized, and their weights are reassigned before being fused to form the final feature vector. Finally, these fused results are passed through the Selective Fusion Module (SFM), followed by the decoder and a small U-Net, to generate the final vessel binary mask.

In the subsequent sections, we introduce the specifics of the selective fusion module and the multi-level attention upsampling module, which constitute components of the proposed segmentation network.

### 2.4 Selective fusion module

The details of the module are displayed in Figure 2. As a crucial component for linking the two feature maps generated by the segmentation network and style transfer network, the SFM is based on knowledge related to feature engineering. Specifically, we propose to select FFA features to replace the broken blood vessel features ensuring that redundant information does not negatively impact segmentation performance. Initially, the network calculates the similarity between the feature map $Z_{1}$ output from the encoder of the segmentation network and the feature map $Z_{2}$ output from the encoder of the style transfer network. The similarity is computed using the Euclidean distance, as shown in Equation 1 below:

$$d=\sqrt{\overset{n}{\underset{i=1}{\sum }}{\left(x_{i}-y_{i}\right)}^{2}}$$ (1)

Where $x_{i}$ and $y_{i}$ denote the pixel values of the two feature maps, respectively, and n denotes the number of pixels of the feature maps. It is worth noticing that the images in this study are rich in information, and based on the limitation of computational resources and time cost, this study carries out partial sampling computation on the feature maps for local similarity comparison. We set a threshold and range of 0.5 to confirm the strength of similarity. Eventually, SFM will fuse the feature map $Z_{2}$ with high similarity to the feature map $Z_{1}$ to generate the fusion feature M . If the similarity between the two feature maps is low, it indicates that $Z_{2}$ does not provide more valuable information for vessel segmentation, and only $Z_{1}$ is passed to the subsequent network layers.

Following the selection and fusion operations, multi-modal information is introduced in this study, which provides higher recognisability for retinal blood vessel segmentation. Additionally, similarity calculation and screening also avoid the interference of irrelevant features on the network when generating FFA images.

### 2.5 Multi-level attention upsampling module

**FIGURE 4:**
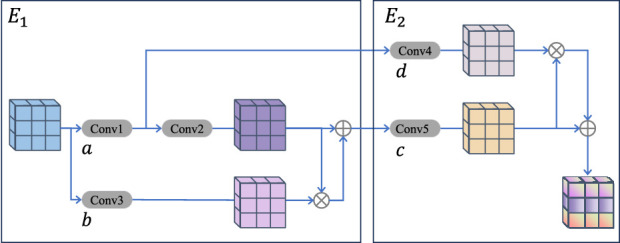
The details of the multi-level attention upsampling module.

As shown in Figure 4, this module is designed to address the issue of vascular information loss during upsampling. The multi-level AUM primarily comprises two compact double convolutional blocks, denoted as $E_{1}$ and $E_{2}$ , which utilize an attention mechanism to capture feature representations at different levels. First, the feature map M generated by the SFM is input into $E_{1}$ , the detailed architecture of which is depicted in Figure 4. $E_{1}$ comprises two convolutional paths of different scales: path a serves as the primary path and encompasses two 7×7 convolutional layers, while path b consists of a single 7×7 convolutional layer. After the feature map M passes through path a and path b , two feature maps $Z_{a}$ and $Z_{b}$ are obtained. We calculate the attention weight $W_{1}$ of $Z_{a}$ with respect to $Z_{b}$ by employing $Z_{a}$ as the query (Q) , key (K) and $Z_{b}$ as value (V) . Subsequently, we perform element-wise multiplication between $Z_{b}$ and the weight $W_{1}$ , and fuse the product with $Z_{a}$ to acquire the upsampling result, integrating feature information across diverse scales. This result is then forwarded to $E_{2}$ , the structure of which is illustrated in Figure 4. $E_{2}$ closely resembles the architecture of $E_{1}$ , with the key difference being the scale of convolution operations. Similar to $E_{1}$ , $E_{2}$ comprises two convolutional paths c and d , with path c serving as the primary path. After passing through both paths, we obtain two distinct feature maps, denoted as $Z_{c}$ and $Z_{d}$ . Subsequently, these feature maps undergo attention computation and fusion steps to yield the decoded result. The process is represented by Equations 2–5:

$$A_{1}=\text{Att}\left(C_{2}\left(C_{1}\left(M\right)\right),C_{2}\left(C_{1}\left(M\right)\right),C_{3}\left(M\right)\right)$$ (2)

$$Output^{*}=C_{2}\left(C_{1}\left(M\right)\right)+A_{1}$$ (3)

$$A_{2}=\text{Att}\left(C_{5}\left(Output^{*}\right),C_{5}\left(Output^{*}\right),C_{4}\left(C_{1}\left(M\right)\right)\right)$$ (4)

$$Output=C_{5}\left(Output^{*}\right)+A_{2}$$ (5)

Where $A_{1}$ , $Output^{*}$ , and $A_{2}$ are the intermediate values computed by the module for the feature map. $A_{1}$ and $A_{2}$ represent attention weight, while $Output^{*}$ represents the output of $E_{1}$ . Output refers to the final output of the module, and Attn represents the attention mechanism. $C_{1}$ , $C_{2}$ , $C_{3}$ , $C_{4}$ , $C_{5}$ correspond five different convolutional layers.

By employing multi-scale convolutions and integrating an attention mechanism, this study enhances retinal blood vessel segmentation with improved structural comprehensiveness. The module excels at fusing feature information across different scales, thereby reducing information loss and omissions during the upsampling process. As a result, it more effectively addresses the challenge of incomplete segmentation of fine retinal blood vessels in UWF fundus images.

## 3 Results

### 3.1 Implementation details

In the experiment, UWF fundus images are preprocessed using the contrast adaptive histogram equalization method to enhance the contrast of the images. The UWF-SEG dataset contains a total of 65 images, with 20 images allocated to the test set and the remaining 45 images utilized for training purposes. The PRIME-FP20 dataset consists of 15 images, with 10 images used for training and the remaining 5 images for testing. To ensure consistency, all images are resized to dimensions of 1024×1024 pixels, with small patches resized to 256×256 pixels. Data augmentation techniques, including random flipping and rotation, are applied during the experiments. The training process spans 200 epochs, enabling the model to converge and effectively capture underlying patterns within the data.

In the experimentation of the proposed method, the model was implemented using PyTorch 3.8 and trained on a workstation equipped with four NVIDIA GeForce RTX 3090 GPUs. A batch size of 5 was used for the UWF-SEG dataset, and 2 for the PRIME-FP20 dataset throughout the training process. The initial learning rate for the vascular segmentation network was set to 0.0004, with a weight decay rate of 0.0005. The Adam optimizer was employed for gradient updates.

### 3.2 Evaluation metrics

To evaluate the performance of the proposed retinal vessel segmentation method, four widely adopted metrics are used in this work, namely,: the Dice Similarity Coefficient (Dice), Sensitivity (Sen), and Balanced Accuracy (BACC). In the evaluation of the method, the sigmoid function was used as the activation function for the final output, and the confusion matrix was calculated (comprising True Positives (TP), False Positives (FP), True Negatives (TN), and False Negatives (FN)) to evaluate the accuracy of the method. The relevant formulas and their significance are as follows:

• Dice Similarity Coefficient (Dice) = 2 × TP/(2 × TP + FP + FN).

• Sensitivity (SEN) = TP/(TP + FN).

• True Positive Rate (TPR) = TP/(TP + FN).

• True egative Rate (TNR) = TN/(FP + TN).

• Balanced Accuracy (BACC) = (TPR + TNR)/2.

Where True Positives (TP) represent the number of samples correctly classified as positive, False Positives (FP) refer to samples incorrectly classified as positive, True Negatives (TN) are the samples correctly classified as negative, and False Negatives (FN) are the samples incorrectly predicted as negative when they should have been positive.

### 3.3 Comparison with state-of-the-art methods

A comprehensive series of comparative experiments were conducted in this section to demonstrate the effectiveness of the proposed method. These experiments included both quantitative and qualitative analyses, evaluating the performance on both public and private test sets. Several state-of-the-art approaches in fundus vessel segmentation were selected for comparison in this study, including CE-Net (Gu et al., 2019), CS-Net (Mou et al., 2021), TransUnet (Chen et al., 2021), SwinUnet (Cao et al., 2022), U-Net (Ronneberger et al., 2015), ResUnet (Xiao et al., 2018) and ConvUNext (Han et al., 2022). All these methods have demonstrated superior performance in fundus image analysis. To ensure the fairness of the experiments, identical image preprocessing and cropping techniques were applied across all methods, and the datasets were divided into the same training test sets.

### 3.4 Performance of vessel segmentation on the private dataset UWF-SEG

The metrics analysis shows that the proposed method outperforms other methods in terms of the effectiveness of retinal blood vessel segmentation in UWF fundus images. Specifically, the M3B-Net method presented in this chapter surpasses other methods across all evaluated metrics (Dice, Sen, and BACC) as demonstrated in Table 1. For example, on the private UWF-SEG dataset, our method achieves improvements of approximately 5.19%, 3.35%, and 1.60% in Dice, Sensitivity, and BACC, respectively, compared to the ConvUNext model (Han et al., 2022). Additionally, our method provides more detailed vascular information, facilitating a finer observation of the retinal vessels. This improvement is primarily attributed to the proposed framework’s focus on enhancing the vascular signal while minimizing the influence of background noise during the reconstruction process.

**TABLE 1:** The vessel segmentation results of different methods on UWF-SEG dataset and FRIME-FP20 datase**t**.

Methods	UWF-SEG			FRIME-FP20		
	*BACC(%)* ↑	*Sen(%)* ↑	*Dice(%)* ↑	*BACC(%)* ↑	*Sen(%)* ↑	*Dice(%)* ↑
U-Net (Ronneberger et al., 2015)	85.34 ± 1.22	71.88 ± 2.62	73.14 ± 0.82	78.90 ± 3.03	57.88 ± 6.08	70.92 ± 4.73
ResUnet (Diakogiannis et al., 2020)	86.54 ± 0.90	74.52 ± 1.71	75.67 ± 0.68	86.74 ± 1.21	73.72 ± 2.43	79.24 ± 1.93
CE-Net (Gu et al., 2019)	87.71 ± 1.66	77.55 ± 3.35	72.64 ± 0.55	88.07 ± 1.02	76.44 ± 2.06	79.88 ± 1.65
CS-Net (Mou et al., 2021)	79.69 ± 3.66	60.18 ± 7.54	68.50 ± 5.54	81.06 ± 2.87	62.24 ± 5.76	73.61 ± 4.49
TransUnet (Chen et al., 2021)	83.95 ± 1.64	68.82 ± 3.39	74.75 ± 0.76	88.12 ± 0.85	76.54 ± 1.72	79.70 ± 1.37
SwinUnet (Cao et al., 2022)	85.24 ± 1.47	71.80 ± 2.98	73.64 ± 0.94	81.65 ± 1.64	63.78 ± 3.39	68.74 ± 1.72
ConvUNext (Han et al., 2022)	88.67 ± 1.27	79.10 ± 2.55	73.35 ± 0.36	91.99 ± 1.31	84.65 ± 3.34	78.06 ± 1.44
Previously Proposed (Li et al., 2023)	89.30 ± 1.22	79.96 ± 2.57	78.13 ± 0.73	92.09 ± 0.59	84.93 ± 1.25	81.97 ± 0.82
Ours	**90.27** ± **1.87**	**82.45** ± **3.80**	**78.54** ± **0.44**	**93.15** ± **0.73**	**87.17** ± **1.53**	**83.02** ± **0.70**

This significant performance improvement can be attributed to the proposed method’s enhanced capability in segmenting fine retinal blood vessels in UWF fundus images. Specifically, the proposed method utilizes cross-modal image style transfer to achieve effective feature enhancement. During the learning process, the selective fusion module enables the segmentation network to select two feature maps based on correlation calculations, which significantly improves the recognition of fine retinal vessels. This approach mitigates the risk of information loss that can occur when directly applying enhancement techniques. Furthermore, given the wide imaging range of UWF fundus images, the study incorporates a local perceptual fusion module and a multi-level attention upsampling module. These modules ensure that global information is effectively integrated during the encoding process while minimizing the loss of vascular details during the decoding stage.

**FIGURE 5:**
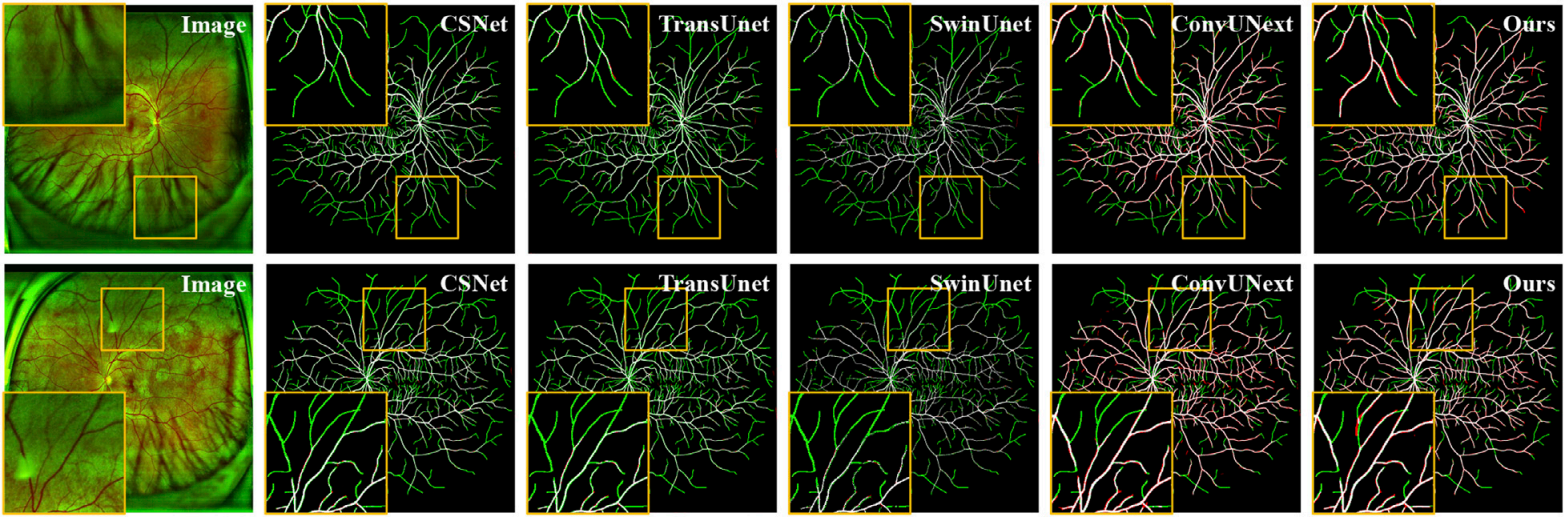
Vessel segmentation results of UWF fundus images by different methods on UWF-SEG dataset, where the white, green, and red represent true positive, false negative, and false positive, respectively.

As shown in Figure 5, the original image from the private UWF-SEG dataset, along with the segmentation results from our method and other comparative approaches, are presented. From the figure, it is evident that TransUnet (Chen et al., 2021), a network structure based on the attention mechanism, is better at capturing both local and global feature relationships than traditional convolutional neural networks. However, the segmentation results exhibit obvious under-segmentation, which indicates that it is more seriously affected by the artifacts and lesion areas in the UWF images during the segmentation process. Additionally, other comparative methods show obvious vessel breaks and unrecognized small vessel ends in the segmentation results. In contrast, our method can more effectively identify the small vessels in UWF fundus images, reducing under-segmentation while also mitigating the over-segmentation issues, as highlighted in the orange boxes in Figure 5.

### 3.5 Performance of vessel segmentation on the PRIME-FP20

Similar to the experimental evaluation metrics for the private dataset, this study also conducts both quantitative and qualitative analyses of all methods on the public PRIME-FP20 dataset. As shown in Table 1, a comparison of the experimental results from our method and other segmentation approaches on the public dataset demonstrates the superior performance of our method. Specifically, on the PRIME-FP20 dataset, our method shows a 3.14% improvement in the Dice score compared to CE-Net (Gu et al., 2019) and outperforms the ConvUNext (Han et al., 2022) by 2.52% in SEN and 1.16% in BACC. These results highlight the effectiveness of our method. Furthermore, our method performs better on the public dataset than on the private dataset, primarily due to the segmentation challenges introduced by lesion regions in the private dataset.

**FIGURE 6:**
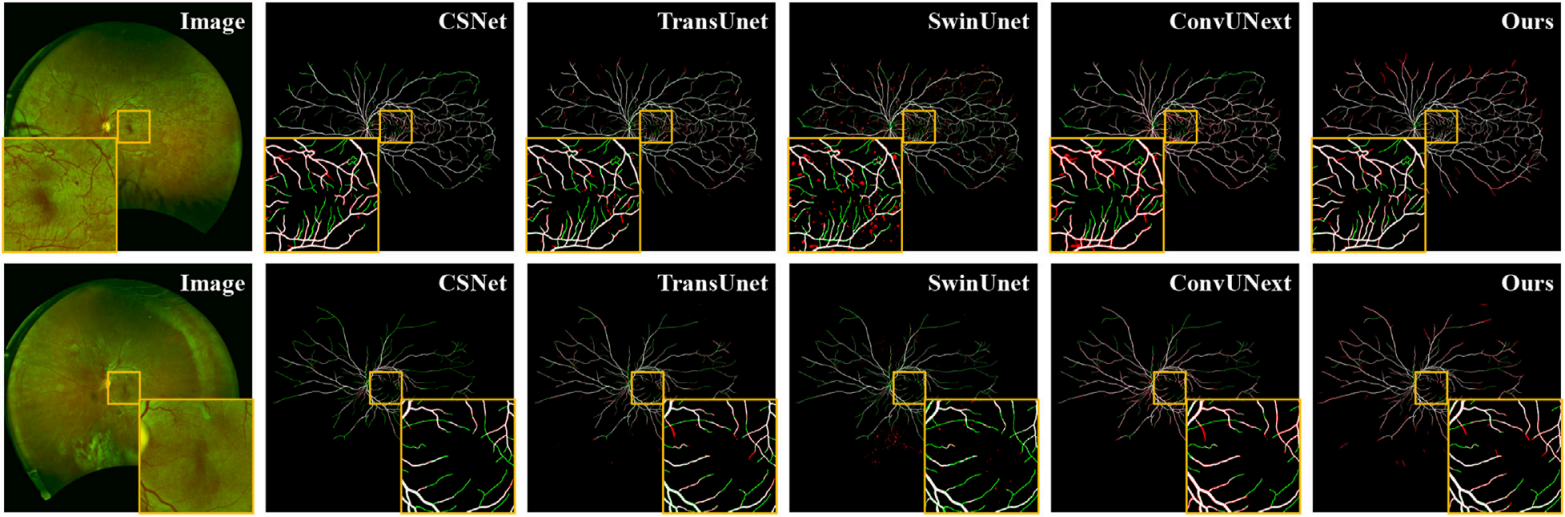
Vessel segmentation results of different methods on PRIME-FP20 dataset, where the white, green, and red represent true positive, false negative, and false positive, respectively.

Additionally, we present both the over-segmentation and under-segmentation results of our proposed method and other comparative methods in Figure 6. It is evident that our method is more effective in recognizing fine vessels and vessels in low-contrast regions. As shown in the orange boxes of Figure 6, although all methods tend to under-segment fine blood vessels near vessel terminals, our method demonstrates superior vessel recognition compared to the others. Furthermore, methods such as SwinUnet (Cao et al., 2022), TransUnet Chen et al. (2021), and CE-Net (Gu et al., 2019) exhibit more pronounced over-segmentation, primarily due to interference from artifacts and lesion areas. In contrast, our method mitigates the impact of these artifacts and noise, thereby reducing the over-segmentation problem.

## 4 Discussion

### 4.1 Ablation studies

To evaluate the performance of the proposed method for retinal vessel segmentation and to further assess the effectiveness of each individual component, this section establishes various baselines for comparison. Extensive experiments are conducted on the publicly available dataset PRIME-FP20, utilizing identical image preprocessing and cropping methods across all baseline methods. As shown in Table 2, LPFM, SFM, and AUM components represent the local perception fusion module, the selective fusion module, and the multi-level attention upsampling module, respectively. The symbol √ denotes the inclusion of a component, while × indicates its exclusion. A comparative analysis of rows 3 to 6 demonstrates that the inclusion of each component significantly improves the results, highlighting their effectiveness. Specifically, incorporating the LPFM component alone improves our method’s BACC, Sen, and Dice metrics by 1.11%, 2.21%, and 0.63%, respectively. Adding the SFM component alone outperforms the baseline method by 2.45%, 4.98%, and 2.09%, respectively. Including the AUM component alone surpasses the baseline method by 1.04%, 2.09%, and 0.96%, respectively. The SFM significantly enhances the segmentation performance of the method by introducing FFA features from the style transfer network. The LPFM enhances the network’s multi-scale perception capability, while the AUM notably improves the results, particularly the sensitivity, indicating that it reduces feature loss during the upsampling process. Additionally, as depicted in rows 7 to 9 of Table 2, the combination of any two out of the three components also enhances performance. Specifically, adding the SFM yields a greater improvement than combining LPFM and AUM, suggesting that FFA features play a crucial role in enhancing retinal blood vessel segmentation in UWF fundus images. Finally, our method achieves better performance by integrating all these components.

**TABLE 2:** The vessel segmentation results of different baseline methods on PRIME-FP20 dataset.

Methods			*BACC(%)* ↑	*Sen(%)* ↑	*Dice(%)* ↑
LPFM	SFM	AUM			
×	×	×	86.37 ± 1.46	73.15 ± 2.97	79.28 ± 1.08
√	×	×	87.48 ± 1.26	75.36 ± 2.56	79.91 ± 1.90
×	√	×	88.82 ± 1.38	78.13 ± 2.83	81.37 ± 0.64
×	×	√	87.41 ± 1.01	75.24 ± 2.06	80.24 ± 1.05
√	×	√	91.49 ± 1.04	83.63 ± 2.14	82.34 ± 0.73
×	√	√	91.05 ± 0.82	82.67 ± 1.67	82.85 ± 0.55
√	√	×	92.60 ± 0.75	85.94 ± 1.53	82.73 ± 0.70
√	√	√	**93.15** ± **0.73**	**87.17** ± **1.53**	**83.02** ± **0.70**

**FIGURE 7:**
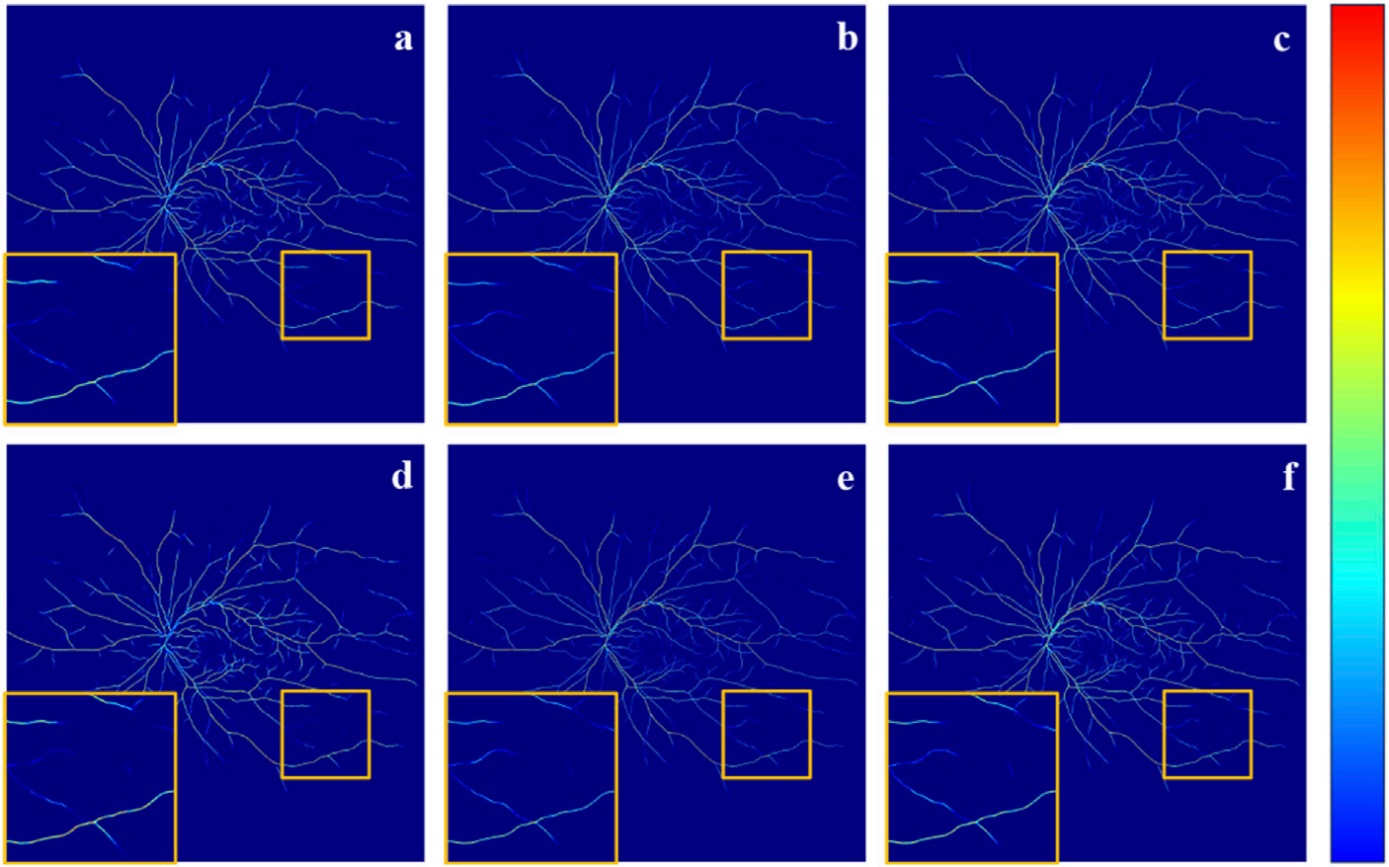
It is the GradCAM visualization of the output from the final layer of the network decoder. Specifically, **(A)** The result obtained without adding any components. **(B)** The result LPFM. **(C)** The result of SFM. **(D)** The result with AUM. **(E)** The result of SFM + AUM. **(F)** The result of LPFM + SFM + AUM.

To further demonstrate the effectiveness of our method and its components, LPFM, SFM, and AUM. We also conducted the GradCAM (Selvaraju et al., 2017) visualization of the output from the final layer of the network decoder, as shown in Figure 7. As observed in Figures 7B, C, and Figure 7D, the orange boxes indicate stronger responses to small blood vessels compared to Figure 7A, particularly in Figure 7C. This demonstrates the effectiveness of the designed components and underscores the advantages of incorporating the FFA modality for low-contrast vessel segmentation. Ultimately, by combining all three components, our method achieves superior vessel segmentation performance.

## 5 Conclusion

We propose an FFA-assisted multi-branch segmentation framework, named M3B-Net, for retinal blood vessel segmentation in UWF fundus images. This framework addresses the challenges of low segmentation accuracy and image boundary artifacts, which are caused by low contrast and the wide imaging range of UWF fundus images. To overcome these issues, M3B-Net integrates a segmentation approach based on style transfer, incorporating a selective fusion module and a multi-level attention upsampling module within the network. Comparative experiments are conducted on two datasets to highlight the superiority of our method, while ablation studies on a public dataset validate the effectiveness of each component. The experimental results demonstrate that our method achieves optimal performance in vessel segmentation of UWF fundus images, successfully overcoming the difficulties associated with segmenting fine blood vessels in these images.

UWF image segmentation enables the precise identification of retinal structures and lesions, facilitating the early detection of diseases such as diabetic retinopathy, age-related macular degeneration, and retinal vein occlusion. Early detection can lead to timely interventions and better patient outcomes. Automated segmentation reduces the reliance on manual annotations, minimizing inter-observer variability and improving diagnostic consistency. This helps ophthalmologists make more accurate and reliable clinical decisions. Therefore, we believe that our method better serves clinical applications by improving retinal vessel segmentation accuracy in UWF fundus images through the integration of the FFA image modality. However, our method may not be very efficient in terms of computational resource consumption. In the future, we will focus on addressing this issue.

## Data Availability

The datasets presented in this article are not readily available because It contains private data. Requests to access the datasets should be directed to jiong.zhang@ieee.org.
